# Luteolin Prevents Cardiometabolic Alterations and Vascular Dysfunction in Mice With HFD-Induced Obesity

**DOI:** 10.3389/fphar.2018.01094

**Published:** 2018-09-25

**Authors:** Daniela Gentile, Matteo Fornai, Carolina Pellegrini, Rocchina Colucci, Laura Benvenuti, Emiliano Duranti, Stefano Masi, Sara Carpi, Paola Nieri, Anna Nericcio, Francesca Garelli, Agostino Virdis, Laura Pistelli, Corrado Blandizzi, Luca Antonioli

**Affiliations:** ^1^Department of Clinical and Experimental Medicine, University of Pisa, Pisa, Italy; ^2^Department of Pharmaceutical and Pharmacological Sciences, University of Padova, Padova, Italy; ^3^Department of Pharmacy, University of Pisa, Pisa, Italy; ^4^Interdepartmental Research Center “Nutraceuticals and Food for Health”, University of Pisa, Pisa, Italy; ^5^Department of Agriculture, Food and Environment, University of Pisa, Pisa, Italy

**Keywords:** obesity, high-fat diet, mice, vascular dysfunction, oxidative stress, inflammation, luteolin

## Abstract

**Purpose:** Luteolin exerts beneficial effects against obesity-associated comorbidities, although its influence on vascular dysfunction remains undetermined. We examined the effects of luteolin on endothelial dysfunction in a mouse model of diet-induced obesity.

**Methods:** Standard diet (SD) or high-fat diet (HFD)-fed mice were treated daily with luteolin intragastrically. After 8 weeks, body and epididymal fat weight, as well as blood cholesterol, glucose, and triglycerides were evaluated. Endothelium-dependent relaxations of resistance mesenteric vessels was assessed by a concentration-response curve to acetylcholine, repeated upon N^w^-nitro-L-arginine methylester (L-NAME) or ascorbic acid infusion to investigate the influence of nitric oxide (NO) availability and reactive oxygen species (ROS) on endothelial function, respectively. Intravascular ROS production and TNF levels were measured by dihydroethidium dye and ELISA, respectively. Endothelial NO synthase (eNOS) and superoxide dismutase 1 (SOD1), as well as microRNA-214-3p expression were examined by Western blot and RT-PCR assays, respectively.

**Results:** HFD animals displayed elevated body weight, epididymal fat weight and metabolic indexes. Endothelium-dependent relaxation was resistant to L-NAME and enhanced by ascorbic acid, which restored also the inhibitory effect of L-NAME, suggesting a ROS-dependent reduction of NO availability in HFD vessels. Moreover, media-lumen ratio, intravascular superoxide anion and TNF levels were increased, while vascular eNOS, SOD1, and microRNA-214-3p expression were decreased. In HFD mice, luteolin counteracted the increase in body and epididymal fat weight, and metabolic alterations. Luteolin restored vascular endothelial NO availability, normalized the media-lumen ratio, decreased ROS and TNF levels, and normalized eNOS, SOD1 and microRNA-214-3p expression.

**Conclusion:** Luteolin prevents systemic metabolic alterations and vascular dysfunction associated with obesity, likely through antioxidant and anti-inflammatory mechanisms.

## Introduction

Obesity represents a major public health and economic problem of global significance that reflects behavioral changes in modern society, including the greater intake of fat-rich food and sedentary lifestyle (World Health Organization [WHO] and Global Health Observatory [GHO], 2014). Obesity is a challenge for global public health, particularly with regard for its implications in several chronic diseases, including cardiovascular diseases (Poirier et al., 2006). Over the last two decades, it has been increasingly recognized that the excessive accumulation of adipose tissue exerts remarkable adverse effects on the vascular system (Poirier et al., 2006; Virdis et al., 2011). In particular, perivascular adipose tissue (PVAT) can directly modulate vascular tone through the secretion of a variety of pro-inflammatory cytokines that in turn give rise to oxidative stress, thereby impairing vascular function (Elmarakby and Imig, 2010; Chatterjee et al., 2013; Virdis, 2016; Reho and Rahmouni, 2017). Of note, endothelial dysfunction, which is an early manifestation of altered vascular homeostasis due to exposure to cardiovascular risk factors, is a predictive index of cardiovascular events in high-risk patients (Lerman and Zeiher, 2005). Indeed, endothelial dysfunction is generally characterized by an increased level of vascular wall oxidative stress, supported mainly by reactive oxygen species (ROS). This leads to a greater NO consumption, resulting ultimately in an impairment of endothelial-dependent vasorelaxation (Furukawa et al., 2004; Heistad, 2006; Munzel et al., 2010; Sena et al., 2013). Data from animal studies have provided compelling evidence that high-fat diet (HFD) leads to both functional and structural changes in the vasculature. In particular, HFD-induced obesity has been reported to alter vascular reactivity (vasoconstriction) and to increase cardiac mass in mice (Calligaris et al., 2013), along with an increase in both inflammation and oxidative stress, strongly implicated in the onset of endothelial dysfunction (Gamez-Mendez et al., 2015).

Currently, there is a great interest in the therapeutic potential of curative plants. Their beneficial effects have been largely attributed to their contents in phenolic compounds, with particular regard for flavonoid derivatives endowed with antioxidant and anti-inflammatory properties (Siasos et al., 2013). Both clinical and animal studies have suggested that antioxidant-rich diets could protect against free radical production and oxidative damage, with a consequent prevention of obesity and related comorbidities (Gentile et al., 2018).

Luteolin, known as 3′,4′,5,7-tetrahydroxyflavone, is one of the most common polyphenolic flavonoids present in several medicinal herbs, fruits and vegetables (Lopez-Lazaro, 2009). Luteolin exerts a variety of pharmacological actions, including antioxidant, anti-inflammatory, antimicrobial, and anticancer effects (Lopez-Lazaro, 2009). Previous *in vitro* and *in vivo* investigations documented its beneficial effects on inflammation and oxidative stress associated with cardiovascular diseases (Qian et al., 2010; Si et al., 2014; Jia et al., 2015).

Recent data suggested also that this flavonoid reduces hepatic steatosis and insulin resistance (El-Bassossy et al., 2013; Xu et al., 2014; Kwon et al., 2015), inhibits cholesterol biosynthesis (Gebhardt, 2002) and increases endothelial NO synthase (eNOS) gene expression (Li et al., 2004). However, the effects of luteolin on obesity-related endothelial dysfunction have not been clarified yet. Therefore, in this study we investigated the impact of luteolin on endothelial dysfunction in a mouse model of diet-induced obesity. Of note, the HFD mouse model is routinely employed to assess the pathophysiological complications associated with obesity, such as cardiovascular disorders and, more specifically, those related to endothelial functions (Rosini et al., 2012). In this respect, data obtained from these mice have a valuable translational potential for human settings.

## Materials and Methods

### Animals

All experiments were approved by the Ethical Committee for Animal Experimentation at the University of Pisa and the Italian Ministry of Health (authorization n°744/2015-PR). Animal care and handling were carried out in accordance with the indications of the European Community Council Directive 2010/63/UE, recognized and adopted by the Italian Government. Experiments were performed on six-week-old male C57BL/6 mice (20–22 g body weight) purchased from ENVIGO Srl (San Pietro al Natisone, Italy). Mice were housed for 1 week in stainless-steel cages in a temperature-controlled (22–24°C) room with a 12:12 h light/dark cycle and 50–60% relative humidity. Mice were allowed access to diets and water *ad libitum*. At the end of *in vivo* procedures, animals were fasted overnight, anaesthetized using chloral hydrate, and sacrificed by cervical dislocation. Blood samples (50 μl) were taken by tail incision and collected in tubes containing heparin to analyze systemic metabolic parameters. Epididymal adipose tissue was removed and weighted. Mesenteric vessels were rapidly excised, cleaned of connective tissue and used for the functional evaluation of endothelium-dependent and -independent relaxation. Other portions of mesenteric arteries were stored at -80°C until needed for further analysis.

### Experimental Procedures

To induce obesity, mice were fed with HFD (60% kcal from fat, TD 06414) for 8 weeks. Control animals remained on SD (18% kcal from fat; TD 2018), which was initially administered to all mice during the first week after their delivery to the laboratory. HFD provided 18.3% kcal as proteins, 21.4% kcal as carbohydrates and 60.8% kcal as fat (5.1 kcal/g), whereas SD provided 24% kcal as proteins, 58% kcal as carbohydrates and 18% kcal as fat (3.1 kcal/g). Subgroups of mice fed with SD or HFD were randomly assigned to one of the four experimental groups (*n* = 5 per group): (1) SD; (2) SD treated with luteolin (10 mg/Kg/day); (3) HFD; (4) HFD treated with luteolin (10 mg/Kg/day). Luteolin (Sigma Chemicals Co., St. Louis, MO, United States) or its vehicle (0.3% carboxymethylcellulose) was administered by oral gavage once daily, starting the first day of HFD administration, and its dose was selected in accordance with a previous report (Liu et al., 2014). Throughout the experimental period, changes in body weight were recorded weekly.

### Blood Cholesterol, Triglycerides, and Glucose Determinations

Total blood cholesterol, triglycerides, and glucose were assayed using MULTICARE IN (Biochemical Systems International Srl, Arezzo, Italy) in accordance with the manufacturer’s protocols.

### Preparation of Small Mesenteric Arteries for Functional Experiments

After dissection, the first branch of mesenteric artery was placed in cold (4°C) physiological salt solution (PSS) containing (in mmol/L): NaCl 120, NaHCO_3_ 25, KCl 4.7, KH_2_PO_4_ 1.18, MgSO_4_ 1.18, CaCl_2_ 2.5, EDTA 0.026, and glucose 5.5, as previously published (Virdis et al., 2003). A second-order branch of the mesenteric arterial tree (≈2 mm in length) was dissected and mounted on 2-glass microcannule in a pressurized myograph, as previously described (Virdis et al., 2007). Vessels were equilibrated for 60 min under constant intraluminal pressure (45 mmHg) in warmed (37°C) and bubbled (95% air and 5% CO_2_) PSS, at pH 7.4. Vessels were considered viable and used if they constricted >70% of their resting lumen diameter in response to an extraluminal application of high-potassium solution (125 mmol/L of KCl) containing 100 μmol/L of noradrenaline (NA).

### Vascular Remodeling and Endothelial Function

Endothelial-dependent and independent vasorelaxation in all experimental groups (n = 5 each group) were defined by the vasodilatory response to cumulative concentrations of Ach (0.001–100 μM) and sodium nitroprusside (0.01–100 μM), respectively. All experiments were performed in vessels precontracted with NA (10 μM). To ensure an equal NA-induced contractility in vessels from SD, SD treated with luteolin, HFD and HFD treated with luteolin mice, we performed preliminary experiments to assess the amount of vasoconstriction induced by increasing NA concentrations (from 1 nM to 100 μM). The results of these experiments showed that NA 10 μmol/L was able to elicit similar contractions among different groups (data not shown), thus such a concentration was selected for functional experiments.

To evaluate the proportion of endothelial-dependent vasodilation due to NO availability, concentration-response curves ACh were constructed after 30 min pre-incubation with the NOS inhibitor N^w^-nitro-L-arginine methylester [(L-NAME) 100 μM, Sigma Co., St. Louis, MO, United States]. To assess the influence of ROS on NO availability, concentration-response curves to ACh were repeated in the presence of the antioxidant ascorbic acid (100 μM, Sigma, 30-min pre-incubation). Finally, to estimate whether ROS generation could impair NO-mediated endothelium-dependent relaxation, ACh was infused during simultaneous incubation with L-NAME and ascorbic acid.

Vessels were then deactivated by perfusion with Ca^2+^-free PSS containing 10 mmol/L EGTA for 30 min. Media thickness and lumen diameter were measured in three different points from each small artery to obtain the media-lumen ratio (M/L), with intraluminal pressure at 45 mmHg (Virdis et al., 2003). Media cross-sectional area (MCSA) was obtained by subtraction of the internal from the external cross-sectional areas using external plus lumen diameters, as previously described (Bruno et al., 2017).

### Detection of Vascular Superoxide Anion Generation

The *in situ* production of superoxide anion from 30 mm frozen mesenteric vessel sections was evaluated at the confocal microscope by means of the fluorescent dye dihydroethidium [(DHE), Sigma], as previously described (Virdis et al., 2015). Three slides per segment were analyzed simultaneously after incubation with Krebs solution at 37°C for 30 min. Krebs-HEPES buffer containing 2 μM DHE was then applied to each section and evaluated under fluorescence microscopy. In the presence of superoxide, DHE undergoes oxidation and intercalates in cell DNA, thus staining the nucleus with red fluorescence (excitation at 488 nm, emission 610 nm). The percentage of arterial wall area stained with the red signal was normalized to the total area examined and quantified using an imaging analysis software (McBiophotonics Image J; National Institutes of Health, Bethesda, MD, United States).

### Assay of TNF in Mesenteric Arteries

Tissue TNF levels were measured with enzyme-linked immunosorbent assay (ELISA) kit (BioSource International, Camarillo, CA, United States), as previously described (Antonioli et al., 2010). For this purpose, mesenteric vessels (30 mg), stored previously at -80°C, were weighed, thawed, and homogenized in 300 μl of ice-cold lysis buffer (200 mM NaCl, 5 m MEDTA, 10 mMTris, 10% glycerine, 1 mM phenylmethylsulfonyl fluoride, 1 mg/ml leupeptin, and 28 mg/ml aprotinin, pH 7.4), and centrifuged at 13,400g for 20 min. Aliquots (100 μl) of the supernatants were then used for assay. Tissue TNF levels were expressed as picogram per milligram of tissue.

### Western Blot Analysis

Samples of mesenteric arteries were homogenized using an assay lysis buffer (Protease Inhibitor Cocktail Tablet, Roche, Indianapolis, IN, United States). Homogenates were spun by centrifugation at 20,000 rpm for 15 min at 4°C. Supernatants were then separated from pellets and stored at -80°C. Protein concentration was determined by the Bradford method (Protein Assay Kit; Bio-Rad, Hercules, CA, United States). Equivalent amounts of protein lysates (50 μg) were separated by 4–10% SDS–PAGE for immunoblotting. After transfer onto a polyvinylidene fluoride membrane, the blots were blocked and incubated overnight with rabbit anti-eNOS [NOS3 (C20): SC-654; Santa Cruz Biotechnology, Santa Cruz, CA, United States] or anti-SOD1 antibodies [SOD1: GTX100554; GeneTex, Irvine, CA, United States]. After repeated washings with Tris-Buffered saline and Tween 20 buffer, appropriate secondary peroxidase-conjugated antibodies (Santa Cruz Biotechnology, Santa Cruz, CA, United States) were added for 1 h at room temperature. Immunoreactive bands were then visualized by incubation with chemiluminescent reagents (Immobilon reagent; Millipore, Billerica, MA, United States) and examined by Kodak Image Station 440 (Celbio, Milan, Italy) for signal detection. To ensure equal sample loading, blots were stripped and reprobed for determination of β-actin by a specific antibody (P5747; Sigma-Aldrich, Milan, Italy).

### Evaluation of Tissue miR-214-3p Expression

Total microRNAs were extracted and purified from frozen mesenteric vessels by using miRNeasy Mini Kit (Qiagen, Germany). Reverse transcription of the extracted miRNAs was performed by the miScript Reverse Transcription Kit (Qiagen, Germany). cDNA was diluted 1:3 in RNase-free water and then qPCRs were performed in triplicate using the miScript SYBR-Green PCR kit (Qiagen, Germany) on the MiniOpticon CFX 48 real-time PCR Detection System (Bio-Rad, Hercules, CA, United States). MiScript Primer Assays specific for mmu-miR-214-3p (MIMAT0000661), mmu-SNORD6 and mmu-RNU6 were obtained from Qiagen. miR-214-3p expression was calculated using Ct method and normalized to the expression of two housekeeping, SNORD6 and RNU6.

### Statistical Analysis

All data sets were represented as the mean ± standard error of mean (SEM). Comparisons of results among groups were carried out by two-way analysis of variance (ANOVA) followed by Newman–Keuls multiple-comparison *post hoc* test. A *P*-value <0.05 was considered as statistically significant. Maximal Ach- and sodium nitroprusside-induced relaxant responses (Emax) were calculated as maximal percentage increments of lumen diameter. All statistical analyses were performed using GraphPad Prism, version 5.0 (GraphPad Software Inc., San Diego, CA, United States).

## Results

### Body and Epididymal Fat Weight

**FIGURE 1:**
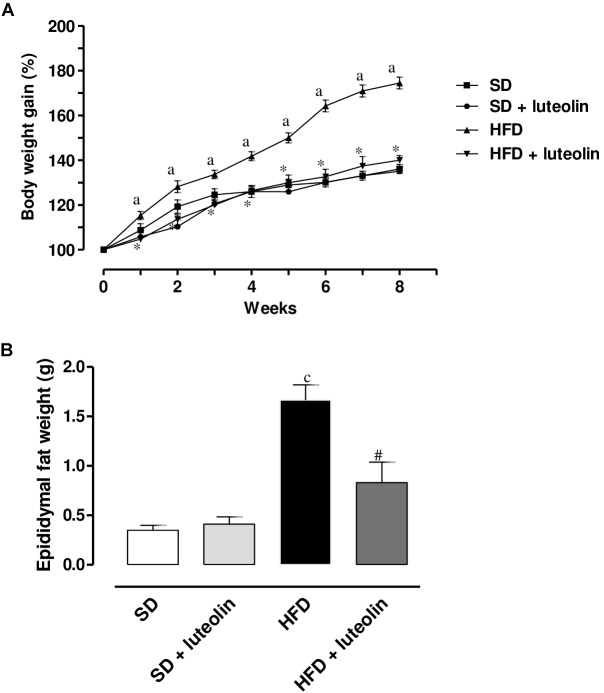
**(A)** Body weight gain (%) in mice fed with SD, SD plus treatment with luteolin (10 mg/Kg/day), HFD or HFD plus treatment with luteolin (10 mg/Kg/day) for 8 weeks. Each point represents the mean ± SEM of five animals. ^a^*P* < 0.05 significant difference vs. SD at the respective week; ^∗^*P* < 0.05 significant difference vs. HFD at the respective week. **(B)** Epididymal fat weight in mice fed with SD, SD plus treatment with luteolin (10 mg/Kg/day), HFD or HFD plus treatment with luteolin (10 mg/Kg/day) for 8 weeks. Each column represents the mean ± SEM of five animals. ^c^*P* < 0.001, significant difference vs. SD; ^#^*P* < 0.001 significant difference vs. HFD. Statistics: two-way analysis of variance followed by Newman–Keuls test.

A significant increase in body weight was observed in HFD as compared with SD mice (**Figure 1A**). Luteolin significantly counteracted the body weight gain in HFD mice (**Figure 1A**). In animals fed with SD, luteolin did not modify the pattern of body weight gain throughout 8 weeks, as compared with control SD animals (**Figure 1A**). In HFD mice the weight of epididymal fat significantly increased as compared to SD animals, and this effect too was counteracted by luteolin (**Figure 1B**). By contrast, epididymal fat weight did not differ when comparing SD and luteolin treated-SD mice (**Figure 1B**).

### Total Cholesterol, Triglycerides, and Glucose Levels

**FIGURE 2:**
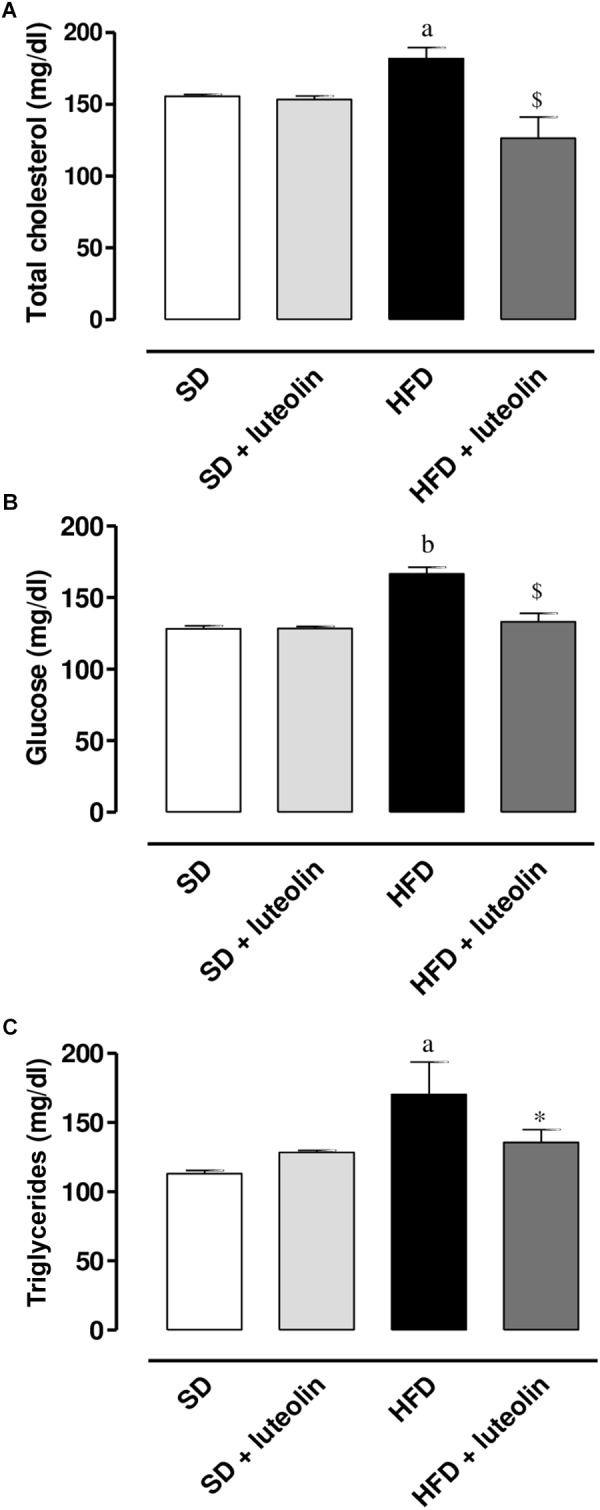
**(A)** Total cholesterol, **(B)** glucose, and **(C)** triglycerides in mice fed with SD, SD plus treatment with luteolin (10 mg/Kg/day), HFD or HFD plus treatment with luteolin (10 mg/Kg/day) for 8 weeks. Each column represents the mean ± SEM of five animals. ^a^*P* < 0.05, ^b^*P* < 0.01 significant difference vs. SD; ^∗^*P* < 0.05, ^$^*P* < 0.01 significant difference vs. HFD. Statistics: two-way analysis of variance followed by Newman–Keuls test.

High-fat diet resulted in a significant increase in total cholesterol, glucose, and triglycerides levels as compared with SD (**Figures 2A–C**). Administration of luteolin to HFD mice prevented partly these metabolic alterations (**Figures 2A–C**). No significant differences in these parameters were observed by comparison of SD and luteolin treated-SD mice at the end of the study (**Figures 2A–C**).

### Endothelium-Dependent Vascular Relaxation and Remodeling

**FIGURE 3:**
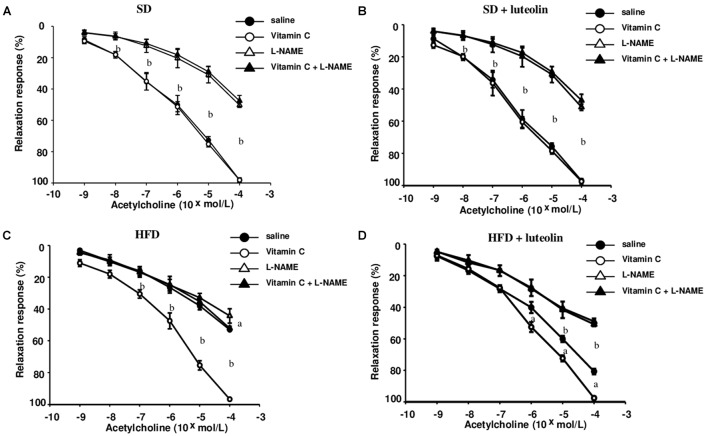
Endothelial-dependent relaxations of mesenteric resistance arteries to acetylcholine in mice fed with SD **(A)**, SD plus treatment with luteolin (10 mg/Kg/day) **(B)**, HFD **(C)**, or HFD plus treatment with luteolin (10 mg/Kg/day) **(D)** for 8 weeks. Each point represents the mean ± SEM of five animals. ^a^*P* < 0.05, ^b^*P* < 0.01 significant difference vs. saline. Statistics: two-way analysis of variance followed by Newman–Keuls test.

In mesenteric vessels from SD, relaxation to ACh was significantly attenuated by L-NAME (100 μM), and not affected by ascorbic acid (100 μM) (**Figure 3A**). Treatment of SD mice with luteolin did not modify the patterns of responses to ACh in mesenteric vessels, in the absence or in the presence of ascorbic acid or L-NAME (**Figure 3B**). Mesenteric vessels from HFD mice showed a reduced vasorelaxation to ACh as compared to SD animals, and also the inhibitory effect of L-NAME on ACh-induced relaxation was significantly attenuated as compared to SD mice. ACh-dependent relaxation in HFD mice was improved by pre-incubation with ascorbic acid, which restored also the inhibitory effect of L-NAME (**Figure 3C**). By contrast, luteolin enhanced the relaxation to ACh in mesenteric vessels from HFD mice and restored the inhibitory effect of L-NAME on ACh-induced relaxation (**Figure 3D**). Endothelium-independent maximal relaxation by sodium nitroprusside was similar in all groups (SD: 96.4 ± 1.6%; SD+luteolin: 97.1 ± 1.1%; HFD: 97.3 ± 1.2%; HFD+luteolin: 96.7 ± 1.8%; P = NS).

**FIGURE 4:**
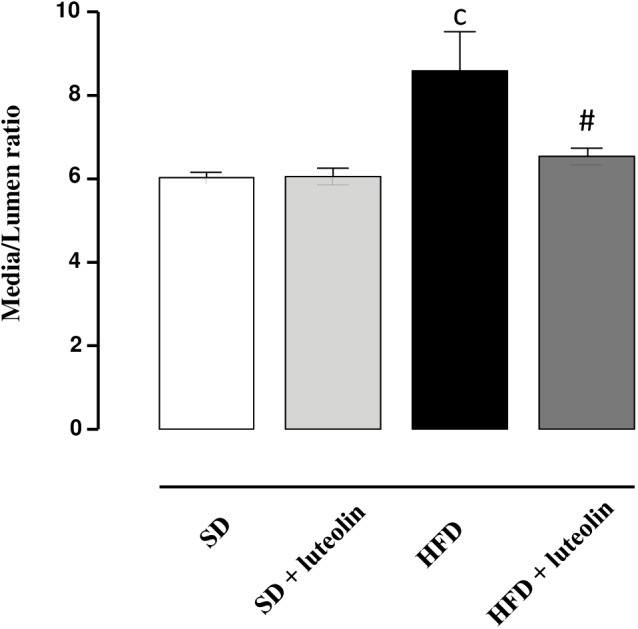
Media-lumen ratio of mesenteric arteries from mice fed with SD, SD plus treatment with luteolin (10 mg/Kg/day), HFD or HFD plus treatment with luteolin (10 mg/Kg/day) for 8 weeks. Each column represents the mean ± SEM of five animals. ^c^*P* < 0.001, significant difference vs. SD; ^#^*P* < 0.001 significant difference vs. HFD. Statistics: two-way analysis of variance followed by Newman–Keuls test.

In keeping with the endothelial function results, media-lumen ratio was significantly increased in HFD mice. The impact of HFD on vascular remodeling and endothelial function was counteracted by luteolin, so that HFD mice supplemented with luteolin showed an endothelial function and media-lumen ratio similar to that of SD mice and significantly improved compared to HFD animals (**Table 1** and **Figure 4**).

**Table 1:** Morphological characteristics and maximal relaxation (%) to acetylcholine ±L-NAME, ascorbic acid or both of mesenteric vessels from the four groups.

	Lumen diameter (μm)	Media thickness (μm)	Acetylcholine	Acetylcholine + L-NAME	Acetylcholine + Ascorbic Acid	Acetylcholine + Ascorbic Acid + L-NAME
SD	262.3 ± 3.8	15.7 ± 0.2	97.1 ± 0.5	51.3 ± 0.8	97.4 ± 0.7	47.1 ± 1.6
SD + luteolin	260.4 ± 4.2	15.6 ± 0.4	96.5 ± 0.6	51.0 ± 0.7	96.3 ± 0.4	50.7 ± 0.8
HFD	217.4 ± 4.8*	18.5 ± 0.2*	53.0 ± 0.2*	44.3 ± 1.8	96.7 ± 0.2	52.1 ± 0.5
HFD + luteolin	237.7 ± 15.8	15.4 ± 0.9	80.7 ± 0.7	49.1 ± 0.8	97.5 ± 0.4	50.9 ± 0.3

*Data are expressed as mean ± SEM. ^∗^*P* < 0.01 vs. other groups Statistics: two-way analysis of variance followed by Newman–Keuls test*.

### Vascular Superoxide Anion Generation

**FIGURE 5:**
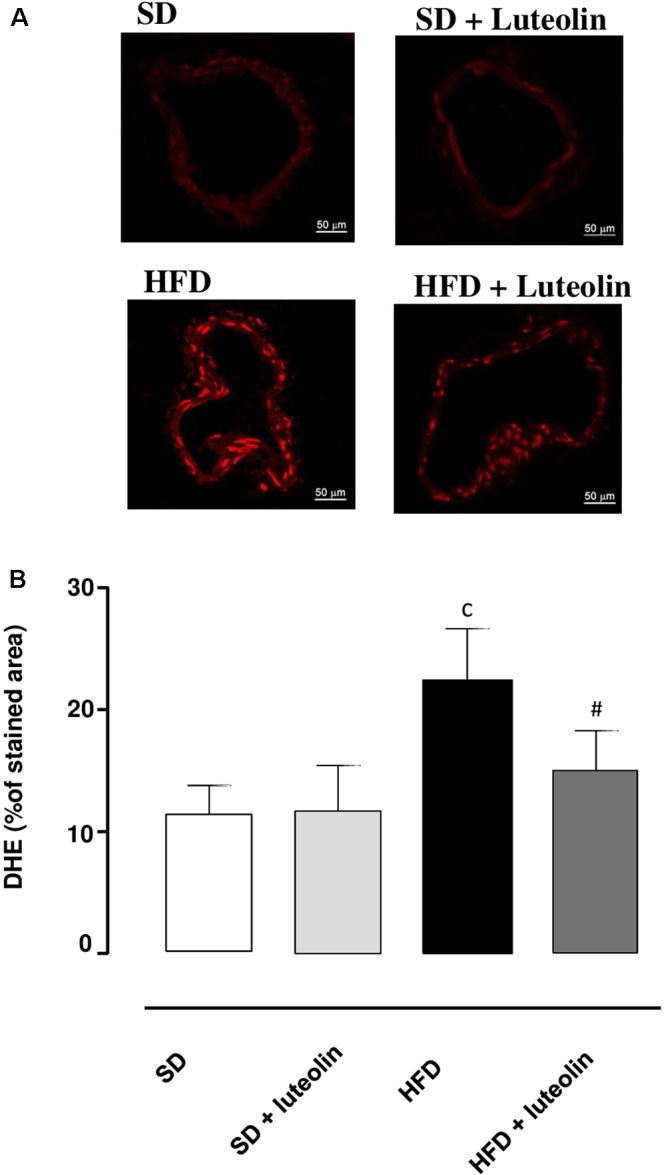
**(A)** Representative dihydroethidium (DHE) staining and **(B)** quantitative analysis (bar graph) of the red signal in mesenteric arteries (magnification ×40) from mice fed with SD, SD plus treatment with luteolin (10 mg/Kg/day), HFD or HFD plus treatment with luteolin (10 mg/Kg/day) for 8 weeks. Each column represents the mean ± SEM of five animals. ^c^*P* < 0.001, significant difference vs. SD; ^#^*P* < 0.001 significant difference vs. HFD. Statistics: two-way analysis of variance followed by Newman–Keuls test.

Dihydroethidium assay revealed a significant increase in superoxide anion production in vessels from HFD mice as compared with SD controls (**Figures 5A,B**). While in SD mice luteolin did not modify superoxide anion generation (**Figures 5A,B**), in HFD mice luteolin treatment counteracted significantly such an increment (**Figures 5A,B**).

### TNF Levels in Mesenteric Arteries

**FIGURE 6:**
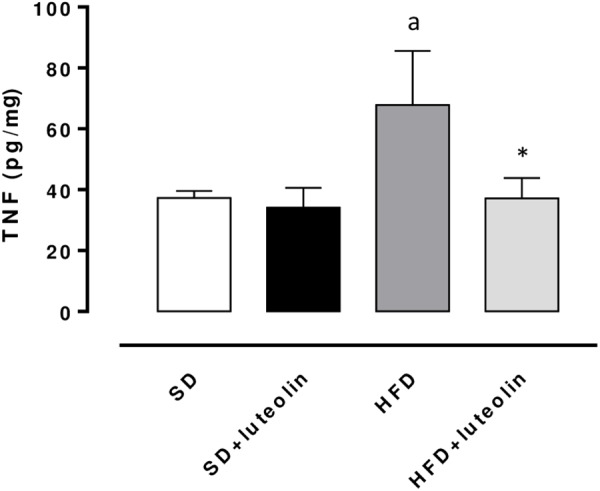
TNF levels in mesenteric arteries obtained from mice fed with SD, SD plus treatment with luteolin (10 mg/Kg/day), HFD or HFD plus treatment with luteolin (10 mg/Kg/day) for 8 weeks. Each column represents the mean ± SEM of five animals. ^a^*P* < 0.05, significant difference vs. SD; ^∗^*P* < 0.05, significant difference vs. HFD. Statistics: two-way analysis of variance followed by Newman–Keuls test.

TNF levels in mesenteric vessels from HFD mice were significantly increased, as compared with SD animals (**Figure 6**). Treatment of HFD mice with luteolin decreased TNF levels toward control values, while the flavonoid did not produce any significant effect in SD mice (**Figure 6**).

### Vascular eNOS and SOD1 Expression

**FIGURE 7:**
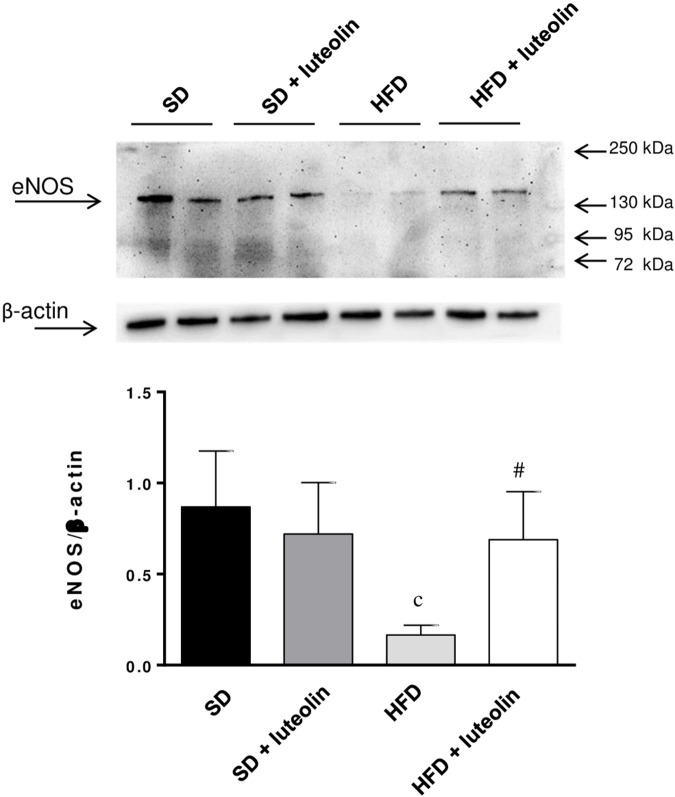
Western blot analysis of eNOS expression in mesenteric arteries from mice fed with SD, SD plus treatment with luteolin (10 mg/Kg/day), HFD or HFD plus treatment with luteolin (10 mg/Kg/day) for 8 weeks. The figure displays representative blots and column graph referring to the densitometric analysis of immunoreactive bands of eNOS normalized to the expression of β-actin. Each column represents the mean ± SEM of five animals. ^c^*P* < 0.001, significant difference vs. SD; ^#^*P* < 0.001 significant difference vs. HFD. Statistics: two-way analysis of variance followed by Newman–Keuls test.

Endothelial NO synthase expression in mesenteric vessels was significantly attenuated in HFD mice, as compared with SD animals (**Figure 7**). In SD mice, luteolin did not modify the expression of eNOS (**Figure 7**). Luteolin administration to HFD mice restored the levels of vascular eNOS expression (**Figure 7**).

**FIGURE 8:**
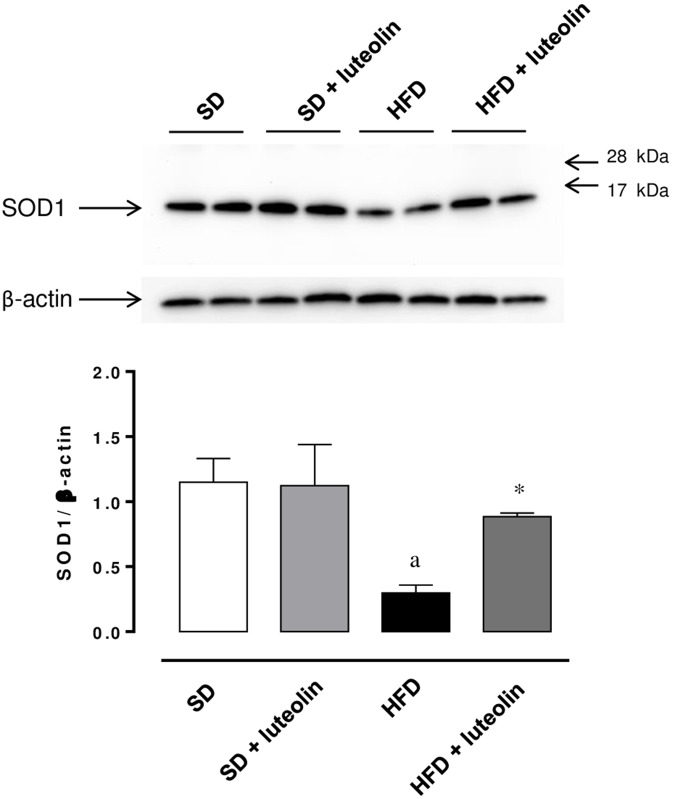
Western blot analysis of SOD1 expression in mesenteric arteries from mice fed with SD, SD plus treatment with luteolin (10 mg/Kg/day), HFD or HFD plus treatment with luteolin (10 mg/Kg/day) for 8 weeks. The figure displays representative blots and column graph referring to the densitometric analysis of immunoreactive bands of SOD1 normalized to the expression of β-actin. Each column represents the mean ± SEM of five animals. ^a^*P* < 0.05, significant difference vs. SD; ^∗^*P* < 0.05 significant difference vs. HFD. Statistics: two-way analysis of variance followed by Newman–Keuls test.

The expression of SOD1 was significantly reduced in mesenteric vessels from mice fed with HFD, as compared with control animals (**Figure 8**). In animals fed with SD, luteolin did not modify the expression pattern of SOD1, while in HFD mice the flavonoid restored SOD1 expression (**Figure 8**).

### miR-214-3p Expression

**FIGURE 9:**
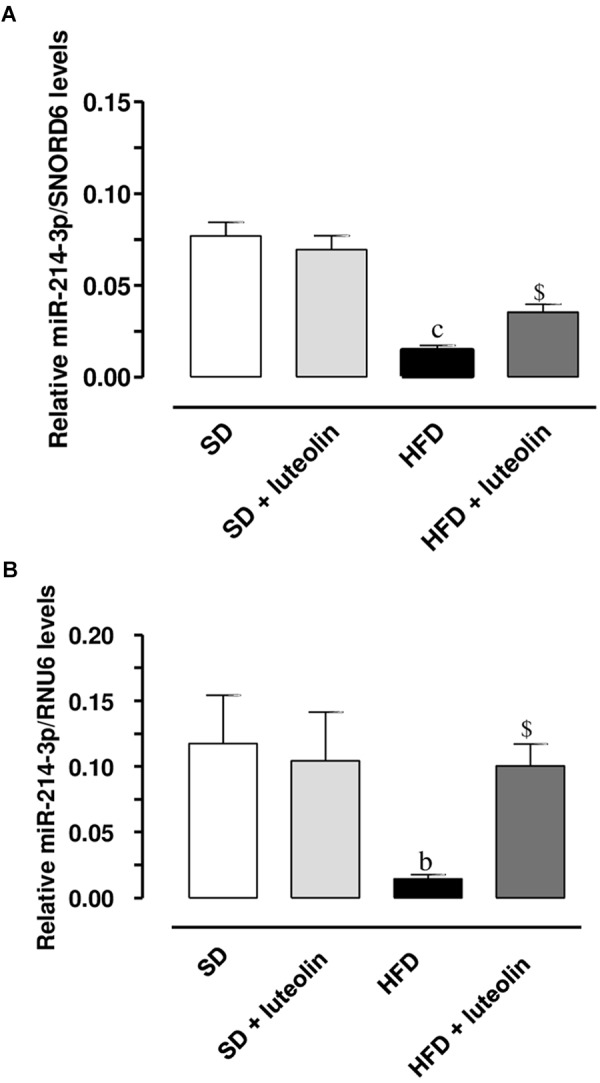
Relative expression of miR-214-3p, normalized vs. the housekeeping genes SNORD6 **(A)** or RNU6 **(B)**, in mesenteric vessels from mice fed with SD, SD plus treatment with luteolin (10 mg/Kg/day), HFD or HFD plus treatment with luteolin (10 mg/Kg/day). Each column represents the mean ± SEM of five animals. ^c^*P* < 0.001, ^b^*P* < 0.01 significant difference vs. SD, ^$^*P* < 0.01 significant difference vs. HFD. Statistics: two-way analysis of variance followed by Newman–Keuls test.

miR-214-3p expression was significantly downregulated in mesenteric vessels from HFD, as compared to levels found in SD mice (**Figures 9A,B**). In SD mice, luteolin did not influence miR-214-3p in mesenteric vessels (**Figures 9A,B**). In HFD mice, luteolin counteracted significantly the decreased expression of miR-214-3p (**Figures 9A,B**). These results were confirmed by normalization vs. the housekeeping genes, SNORD6 and RNU6.

## Discussion

This study provides the first experimental evidence that luteolin is able to prevent weight gain, metabolic alterations, endothelial dysfunction and vascular remodeling related to HFD-induced obesity. We show also that the benefits of luteolin on endothelial function and vascular remodeling are likely mediated by its anti-oxidant and anti-inflammatory properties, resulting in a preserved NO availability within the vascular wall. Obesity is a major risk factor for health, as it is associated with a significant increase in morbidity, mortality and economic costs for healthcare systems (Berrington de Gonzalez et al., 2010). Cardiovascular disease represents the most important complication associated with obesity and the development of endothelial dysfunction combined with vascular remodeling are the earliest manifestations of an altered vascular homeostasis, involved in the initiation, evolution, and complications of cardiovascular disorders. Thus, if the results provided by our study could be translated to humans, luteolin might become an important tool for treatments aimed at preventing obesity and its related complications. Of note, besides the beneficial effects exerted by luteolin on endothelial function, it is likely that its anti-inflammatory and antioxidant effects could take a part in the improvement of metabolic syndrome, and particularly the diabetic condition, associated with HFD, as also previously reported (Deqiu et al., 2011; Bansal et al., 2012).

The experimental model of diet-induced obesity has been shown to be suitable for investigations on the pathophysiology of complications associated with obesity, such as metabolic syndrome and cardiovascular disorders and, more specifically, those related to endothelial function (Rosini et al., 2012). Furthermore, among the obesogenic diets, HFD contributes significantly to weight gain, the consequent development of obesity, and related disorders (Mozaffarian et al., 2011). Our results are in keeping with previous observations (Antonioli et al., 2017), confirming that mice fed with HFD for 8 weeks developed a marked increase in body and epididymal fat weight associated with an elevation of total cholesterol, triglycerides, and glucose, thus corroborating further the suitability of this experimental model.

Interestingly, our results from functional experiments indicated that mesenteric small arteries from HFD animals display altered endothelium-dependent relaxations due to a reduced NO availability. Such a deficiency is likely to depend on an increased intravascular ROS generation, as documented by the restored response to ACh after preincubation with ascorbic acid, and the increased DHE fluorescence in the vascular wall from HFD as compared to SD mice. In particular, superoxide anion overproduction has been previously shown to take a significant part in the development of endothelial dysfunction, through the reaction with NO and subsequent production of peroxynitrite (Fukai and Ushio-Fukai, 2011). In our experiments, we observed that the expression of SOD1, a key enzyme involved in the neutralization of superoxide anions (Fukai and Ushio-Fukai, 2011), was significantly decreased in mesenteric vessels from HFD-fed mice, thus suggesting that the endothelial dysfunction elicited by HFD could depend also on the reduced ability of vascular tissues to scavenge superoxide anions. In agreement with our results, recent reports have documented a marked endothelial dysfunction associated with a significant vascular pro-oxidant condition in HFD animals (Ketonen et al., 2010; Heinonen et al., 2014; Toral et al., 2015), as well as a decreased SOD1 scavenging activity (Mo et al., 2018). Moreover, evidence from clinical studies, showing a correlation of obesity with compromised vascular function, is available also. Romero-Corral et al. (2010) showed that an 8-week overfeeding period increased visceral adiposity and resulted in an impairment of endothelial function in humans. The authors observed, as a proof-of-concept, that the impairment of endothelial function was reversible when the visceral fat was reduced by pre-planned caloric restriction (Romero-Corral et al., 2010). Virdis et al. (2011) have shown that vessels from obese individuals displayed a blunted endothelium-dependent relaxation, caused by a reduced NO availability. Of note, alterations were reversed by either a superoxide scavenger or a TNF inhibitor, suggesting a key role of oxidative stress as well as of vascular inflammation (Virdis et al., 2011). With regard to the latter aspect, our data on TNF levels in mesenteric arteries from HFD mice confirmed a condition of obesity-related low-grade inflammation that may lead to an increase in oxidative stress, thus exerting detrimental effect on endothelial function.

In view of the pathophysiological role of eNOS in obesity, whole-body metabolism and vascular function (Sansbury and Hill, 2014), we evaluated eNOS levels within the vascular wall. As expected, HFD mice showed a lower eNOS protein expression as compared to SD mice. However, such a decrease does not seem to take a significant part in the altered endothelium-dependent relaxation observed in HFD animals, since the *in vitro* antioxidant effect exerted by ascorbic acid was able *per se* to restore the vasorelaxant effect of ACh in HFD mice. Thus, these data suggest that, under antioxidant conditions, the residual level of eNOS expression in HFD vessels is able to ensure an NO production sufficient to elicit a complete vasodilation. Nevertheless, the decreased expression of eNOS in HFD mice could be strongly related to the pathophysiology of obesity and metabolic function. In support of this concept, it is documented that eNOS abundance is decreased remarkably in obese and diabetic humans and rodents, thus suggesting its central role in regulating body composition (Sansbury and Hill, 2014). Moreover, evidence from an *in vivo* study showed that an increased eNOS expression prevented the obesogenic effects of HFD (Sansbury et al., 2012).

Among the different pathways, which could contribute to vascular remodeling, we decided to investigate on miRNAs, since they are increasingly recognized as key regulators of important cardiac physiological processes, but are implicate also in the pathological progression of cardiovascular diseases. The significant downregulation of miR-214-3p, as observed in mesenteric arterioles from HFD mice, might be important in mediating the impact of HFD on endothelial function. As documented by other authors, miR-214 undergoes modulation under several pathological conditions, and it contributes to the pathogenesis of various human disorders, including cancer and cardiovascular diseases (Zhao et al., 2017). Moreover, miR-214 expression was reported to be decreased in senescent endothelial cells, in which a lowered eNOS protein level was documented as well (Rippe et al., 2012). The inverse correlation between miR-214 and eNOS expression, reported, by contrast, by Chan et al. (2009) might be related to the “young” population of endothelial cells. In this regard, Zhao et al. (2017) had previously suggested that miR-214 might have opposite effects on “young” vs. “mature” endothelial cells with regard for angiogenesis (inhibition vs. promotion).

Endothelial dysfunction, being a functional reversible alteration, represents an attractive target for prevention strategies against cardiovascular diseases. Most recent studies on the management of obesity and related disorders have been focused on the potential role of plant compounds, with particular regard for polyphenols. In this context, luteolin, a food-derived flavonoid natural compound, is particularly attractive. Luteolin is present in medicinal plants as well as in some vegetables and spices, and it has received wide attention for its antitumor, antioxidant, and anti-inflammatory effects as well as its capacity to improve vascular functions in *in vitro* studies (Pietta, 2000).

Based on the above considerations, the second part of the present study was dedicated to investigate the effects of luteolin in counteracting the endothelial dysfunctions associated with HFD-induced obesity. Notably, in mice fed with HFD, luteolin was effective in counteracting the increase in body and epididymal fat weight, as well as in reducing the elevations of blood total cholesterol, triglycerides, and glucose. These findings support previous observations highlighting an ameliorative effect of luteolin in obese mice in terms of weights of body, liver, white adipose tissue and elevated plasma lipids (Liu et al., 2014; Kwon et al., 2015).

Of interest, in our experiments, luteolin was effective also in mitigating vascular dysfunction. In particular, vessels from HFD mice treated with luteolin showed an improved relaxation to ACh, which became sensitive to L-NAME, while ascorbic acid was no longer able to affect the vascular response to ACh. The demonstration that, in the presence of luteolin, L-NAME counteracted the relaxation to ACh, an effect not exerted under baseline conditions, implies that NO availability was restored after treatment with this flavonoid. It is worth noting that, following luteolin administration, the restored inhibiting effect of L-NAME on ACh was similar to that observed after acute exposure to ascorbic acid at baseline, thus suggesting an antioxidant property of luteolin. In our results, this concept is strengthened also by the luteolin ability of attenuating the intravascular superoxide excess in HFD mice. Of note, this antioxidant effect could also derive from the ability of luteolin to counteract the HFD-induced decrease in SOD1 expression in mesenteric vessels. Our data are supported by previous observations, indicating luteolin as a vascular protective agent that stimulates NO-dependent vascular dilatation by acting on vascular endothelial cells (Si et al., 2014). Moreover, luteolin was found to improve the ventricular function and coronary flow throughout reperfusion, to increase the cardiac tissue viability and enhance the manganese superoxide dismutase activity in diabetic rats (Yang et al., 2015), as well as to protect rat mesenteric arteries from injury by superoxide anion (Ma et al., 2008).

In our experiments luteolin reduced TNF levels in mesenteric arteries from HFD mice, thus suggesting an anti-inflammatory activity. In agreement with our data, the ability of luteolin in mitigating pro-inflammatory processes has been previously described in the prevention of TNF-induced vascular inflammation in both endothelial cells and mice (Jia et al., 2015). In particular, it was shown that luteolin attenuated vascular inflammation by suppressing TNF-stimulated expression of chemokines and adhesion molecules, as well as preventing the activation of nuclear factor (NF)-κB signaling in both experimental models (Jia et al., 2015). Moreover, in an another study, luteolin was found to reduce mRNA expression of pro-inflammatory cytokines such as TNF and interleukin (IL)-6 in palmitic acid-induced IκB kinase (IKKb)/NF-kB activation in human umbilical vein endothelial cells (HUVECs) (Deqiu et al., 2011).

In the present study, luteolin administration to HFD mice restored the levels of eNOS expression within vascular wall, in keeping with previous evidence, showing that luteolin prevented the decrease of eNOS expression induced by palmitic acid in HUVECs (Deqiu et al., 2011). However, the effects of luteolin on eNOS expression observed in HFD mice, does not seem to account significantly for the beneficial effects of the flavonoid on functional endothelium-dependent relaxation of mesenteric arteries. Indeed, functional data showed that the relaxation of mesenteric arteries was not restored by luteolin, while it is reestablished by ascorbic acid, suggesting an involvement of oxidative stress. Therefore, the ability of luteolin to increase eNOS expression could take a significant part in mediating its anti-obesogenic effects (Sansbury et al., 2012; Sansbury and Hill, 2014), rather than an active involvement in counteracting endothelial dysfunction.

Finally, in our experiments, luteolin was able to counteract the decreased expression of miR-214-3p in mesenteric vessels from HFD mice, likely due to its ability in regulating the inflammatory changes induced by HFD. Moreover, our observation supports the evidence, made by Rippe et al. (2012) that parallel changes in eNOS and miRNAs expression in endothelial cells can be involved in vascular dysfunction.

## Conclusion

The present results suggest that luteolin can prevent systemic metabolic alterations and vascular dysfunction associated with obesity, likely through both antioxidant and anti-inflammatory properties.

## Author Contributions

CB, MF, LA, RC, DG, LP, PN, AN, FG, and AV contributed to the experiment design. DG, CP, LB, ED, SM, and SC contributed to the acquisition and analysis of data. CB, MF, LA, RC, LP, PN, and AV obtained the funding. DG, MF, LA, and CB wrote the manuscript. All authors have read and approved the final manuscript.

## Conflict of Interest Statement

The authors declare that the research was conducted in the absence of any commercial or financial relationships that could be construed as a potential conflict of interest.
